# The prevalence and persistence of aberrant promoter DNA methylation in benzene-exposed Chinese workers

**DOI:** 10.1371/journal.pone.0220500

**Published:** 2019-08-05

**Authors:** Jingchao Ren, Jun-peng Cui, Mengkai Luo, Huan Liu, Pengfei Hao, Xiao Wang, Guang-hui Zhang

**Affiliations:** 1 Henan International Collaborative Laboratory for Health Effects and Intervention of Air Pollution, Medicine, School of Public Health, Xinxiang Medical University, Xinxiang, China; 2 Xinxiang Center for Disease Control and Prevention, Xinxiang, China; University at Buffalo - The State University of New York, UNITED STATES

## Abstract

Aberrant DNA methylation patterns are common in cancers and environmental pollutant exposed subjects. Up to date, few studies have examined the aberrant DNA methylation patterns in benzene exposed workers. We recruited 141 benzene-exposed workers, including 83 benzene-exposed workers from a shoe factory in Wenzhou and 58 workers from a painting workshop in Wuhu, 35 workers in Wuhu were followed from 2009 to 2013, and 48 indoor workers as controls from Wenzhou. We used high-resolution melting (HRM) to quantitate human samples of DNA methylation in long interspersed nuclear element-1 (LINE-1), (6)-methylguanine-DNA methyltransferase (MGMT), and DNA mismatch repair gene human mutator L homologue 1 (hMLH1). AML-5 cells were treated with benzoquinone (BQ) and hydroquinone (HQ), and the promoter methylation of MGMT and hMLH1 was detected using the bisulfite sequencing PCR method. The degree of LINE-1 methylation in benzene-exposed workers was significantly lower than that of the controls (*p*<0.001), and the degree of MGMT (*p*<0.001) and hMLH1 (*p* = 0.01) methylation was significantly higher than that of the controls. The in vitro study validated the aberrant hypermethylation of hMLH1 after treatment with BQ. Among the cohort workers who were followed from 2009 to 2013, the LINE1 methylation elevated in 2013 than 2009 (*p* = 0.004), and premotor methylation in hMLH1 reduced in 2013 than 2009 (*p* = 0.045) with the reduction of the benzene exposure. This study provides evidence that benzene exposure can induce LINE-1 hypomethylation and DNA repair gene hypermethylation.

## 1 Introduction

Benzene is a ubiquitous environmental and occupational pollutant that has been classified as a Group 1 carcinogen by IARC (IARC 1982). It is well known that chronic poisoning caused by benzene still occurs in both Western countries and in China, and it remains significant as a high number of leukemia patients has been reported in recent years [1–3]. However, the mechanism of its carcinogenesis is still unclear, and the hallmarks of early damage caused by benzene are still not well illuminated.

Recently, DNA methylation has provided new insights in the study of cancer as abnormalities of DNA methylation may be the key to initiating tumorigenesis [4]. It is generally accepted that cancer arises from genetic and epigenetic errors induced by environmental pollutants. The methylation of cytosines allows the encoding of heritable epigenetic information directly onto the DNA while leaving the DNA nucleotide sequence intact, and its role in carcinogenesis has become a topic of considerable interest in the last few years [5]. Furthermore, more attention is being paid to environmental pollution-induced alterations of DNA methylation, including global hypomethylation, gene-specific hypermethylation or the hypomethylation of specific genes.

DNA methylation changes in the peripheral blood have been considered to have great potential as a source of locus-specific biomarkers that can be used for the early detection and monitoring of environmental pollution [6, 7]. This promise is considerable and has been fulfilled by the use of alterations of DNA methylation in populations exposed to pollutants such as cadmium[8], and polycyclic aromatic hydrocarbon[9]; however, a new and more reliable, repaid and sensitive method for promoter methylation analyses in clinical samples is needed. High-resolution melting (HRM) relies on the precise monitoring of changes in fluorescence as a DNA duplex melts and is capable of analyzing homogeneous methylation in a semi-quantitative manner[10–12].

In the present study, we focus on the global DNA methylation surrogated by the level of long interspersed nucleotide element-1 (LINE–1) methylation and on the promoter regions in DNA repair genes, indicated as O6-methylguanine-DNA methyltransferase (MGMT), and human mismatch-repair gene (hMLH1). LINE-1 sequences comprise approximately 20% of the genome and are directly associated with multistep carcinogenesis and aggressive cancers with poor prognoses [13–15]. The promoter region of methylation in either hMLH1 or MGMT is known to cause high-degree microsatellite instability (MSI-H) and guanine-to-adenine mutations in KRAS, TP53 and PIK3CA[16, 17]. Those are also the most widely studied biomarkers of repetitive sequences within the context of DNA methylation change and environmental carcinogen exposure.

While DNA methylation in tumors has been extensively studied, little is known about aberrant DNA methylation, especially the DNA repair genes, in normal benzene-exposed workers. In this study, benzene-exposed workers from two cities were examined, and some were followed from 2009 to 2013. The cytokinesis-block micronucleus (CBMN) assay and hemograms of peripheral blood were conducted. We focused on both the variation in DNA methylation in benzene-exposed workers over the two time periods and on the use of in vitro AML-5 cells treated with benzoquinone (BQ) and hydroquinone (HQ) to detect DNA methylation.

## 2. Methods

### 2.1 Study population

**Fig 1:**
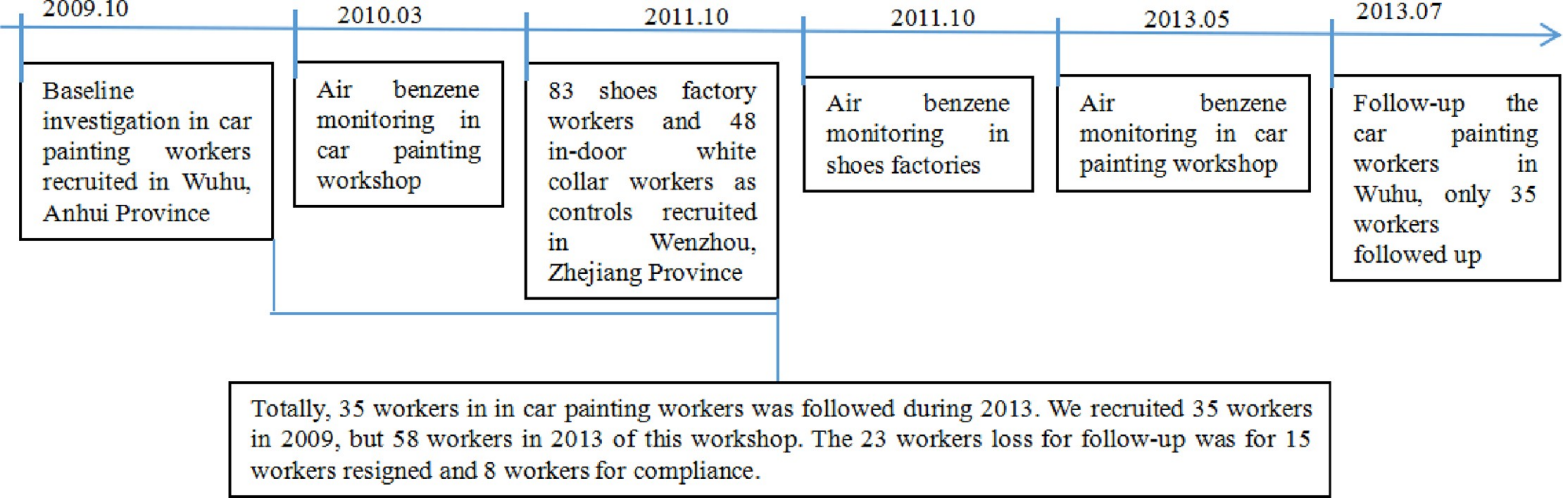
Flow chart of design for the human study.

The study protocol was approved by the Research Ethics Review Board of the School of Public Health, Fudan University and Xinxiang medical university. The occupational epidemiological survey was approved by School of Public Health, Fudan University, and the in vitro work was approved by Xinxiang medical university. Written informed consent was obtained from each individual during the interview. Fig 1 showed the flow chart of design for the human study. On the basis of employment records, 58 BZ-exposed workers were recruited from a painting workshop in Wuhu, Anhui Province, China, during routine medical evaluations in 2013. In Zhejiang Province, 83 benzene-exposed workers from a shoe factory who used similar materials and production work flows were recruited during routine medical evaluations in Wenzhou, Zhejiang province (S1 Table). Each subject responded to an interviewer-administered questionnaire, which consisted of demographic characteristics, medication use, smoking and drinking habits, occupational history, and independence symptoms. Workers exposed to benzene for at least 1 year were selected if they had completed the detailed questionnaires. Additionally, 48 indoor workers from Zhejiang served as controls. The participants’ data are shown in Table 1. Both groups were ethnically Han Chinese. Furthermore, 35 benzene-exposed workers (29 men and 6 women, aged 25 to 44 years old) were recruited for prospective follow-up from 2009 to 2013 (S2 Table).

**Table 1:** Basic demographic characteristics of the study subjects.

Parameters	Controls 48 (%)	Painting workers in 2009 (35)		Painting workers in 2013 (58)		Shoes workers (83)	
		Number (%)	*p*^*a*^	Number (%)	*p*^*a*^	Number (%)	*p*^*a*^
Gender							
Male	25 (52)	29 (83)		50 (86)		39 (47)	
Female	23 (48)	6 (17)	0.005	8 (14)	<0.001	44 (53)	0.352
Smoking							
Non-smoker	42 (88)	24 (69)		35 (60)		66 (80)	
Smoker	4 (8)	11 (31)	0.019	23 (40)	<0.001	17 (20)	0.065
Drinking							
Non-alcohol user	26 (54)	13 (37)		19 (33)		59 (71)	
Alcohol user	22 (46)	22 (63)	0.499	39 (67)	0.163	24 (29)	0.033
Age: Median (Mix, Max)	45(25, 79)	35 (21,44)	0.001	28 (21, 44)	<0.001	30 (18, 51)	<0.001
Air benzene (mg^3^/m^3^)	---	0–3.4 in 2010	---	No benzene in 2013^b^	---	2.6–57.0	---

^a^ p- values were got from the comparison of the controls and car painting workers or shoe workers. Age were carried out by Mann-Whitney U test.

^b^ We didn’t find the benzene in the car painting worksites in Wuhu city in the year of 2013. This perhaps for the detection limit was high as 0.6 mg/m^3^. And the air benzene concentration in normal environment of the control.

After obtaining informed consent, 5 ml of anti-coagulated peripheral blood was collected from each subject. The blood collection was conducted with empty stomach in morning during 8–10 a.m. For car painting workers, the questionnaire surveys and blood collection occurred in the years 2009 and 2013.

### 2.2 Assessment of benzene exposure

The painting workshop was chosen for the analysis of the airborne benzene levels. We sampled 0.1 L/min for 15 minutes three times a day at 9:00, 11:00, and 16:00 in Wenzhou and Wuhu using the QC-4 air sampler (Yinhekeji, Jiangsu, China) according to the Chinese standards (GBZ159-2004). The samples collected in 2010 in car painting worksites were taken five months after the interviews that were conducted in 2009; and the worksites were followed to detected in 2013. The car factory improved the technique to reduce the air benzene concentration after the blood collection in 2009 and air sampling in 2010, at the end of 2010. While the worksites in shoe factories were taken in 2011 while the conduction worker sampling. Air samples were also collected from different worksites at the plant three times during the study. The samples were analyzed using GC7890 I gas chromatography (AoSong Scientific Instruments, Shanghai, China) with a flame ionization detector (GC-FID) according to the Chinese standard (GBZ/T 160.42–2007).

### 2.3 Cytokinesis-block micronucleus assay

The CBMN method was used as described by Fenech [18, 19]; this method was also described in our previous study [20]. In brief, 0.5 ml of heparinized whole blood was cultured and incubated at 37°C in 4.5 ml of medium (RMPI 1640) with phytohemagglutinin (PHA) stimulation for 44 h; then, Cytochalasin-B (Sigma, USA) was added at a final concentration of 6 μg/ml, and the cultures were harvested 28 h later. A 3:1 mixture of methanol and acetic acid was used to fix the lymphocytes before they were transferred to slides. The MN frequency was scored using 1,000 bi-nucleated (BN) lymphocytes with well-preserved cytoplasm. Samples from the exposed and control workers were scored blindly.

### 2.4 Hemogram detection

Peripheral blood hemograms are a compulsory component of the medical examination for workers exposed to benzene in China. An automated hematology analyzer (XE-2100, Sysmex, Japan) was used to detect the hemograms of both the exposed and the control group members.

### 2.5 DNA extraction and bisulfite conversion

DNA was extracted from peripheral blood leukocytes using the DNA blood isolation kit (Life Feng Biotechnology Co., Shanghai, China) according to the manufacturer’s instructions and was frozen at -80°C. Fully CpG-methylated human genomic DNA was obtained from Fermentas (Thermos Scientific, Fermentas), and unmethylated control DNA was obtained commercially (Qiwu, Shanghai, China).

For the DNA standards and participant samples, whole DNA was quantified on a NanoDrop ND-2000 UV-Vis spectrometer (Thermo Scientific, USA). An Epitect Bisulfite Conversion Kit (Qiwu, Shanghai, China) was used to deaminate cytosine to uracil but not to 5-methyl-cytosine. The Epitect Bisulfite Conversion Kit was used to convert 500 ng DNA according to the manufacturer's protocol, and the modified DNA was diluted at a concentration of 20 ng/μl. The primers for LINE-1 were as previously reported [21], including 141 bp amplicon within a total of 8 CpG dinucleotides; the 8 CpG sites of interest have been validated as representative of global DNA methylation status [22]. The primers for MGMT in the promotor region were previously described [10]. The hMLH1 primers were as follows: F- TTGCGGGAGGTTATAAGAGTAGGGTT, R- CCGAAACCGAACTTATATACCTCTACTAAAATAAT. The 132-bp amplification included a total of 5 CpG dinucleotides between the primers and ranged from -248 to -77 in the promoter region, which was the range that was most correlated with the expression of hMLH1[23].

We performed PCR in 0.2-mL tubes with a final reaction volume of 20 μL containing 200 nmol/L of each primer, 200 μmol/L of each dNTP, 5 μmol/L SYTO 9 (Invitrogen, Life Technologies), 2.5 mmol/L MgCl_2_, 0.5 U HotStarTaq DNA polymerase (Qiagen) according to a previous study[24]. PCR was performed with a ViiA™ 7 Real-Time PCR system (Life Technologies, USA) as follows: 1 cycle of 95°C for 1 min, 50 cycles of 95°C for 10 sec, 55°C for 10 sec and 72°C for 15 sec. This was immediately followed by a hold at 95°C for 1 min, 72°C for 1 min and an HRM step from 72 to 95°C, increasing by 0.025°C per sec and holding for 1 sec after each stepwise increment.

### 2.6 Cell culture and treatment

AML-5 cells were purchased from Cobioer Biosciences in Najing, China, and cultured according to instructions (Medium: 70% α-MEM, 20% FBS, 10 ng/ml GM-CSF). The cells were treated in the logarithmic growth phase with benzoquinone (BQ) (Sigma) and hydroquinone (HQ) (Sigma) at final concentrations of 0 and 40 μM, respectively, and the cells were harvested after 72 h of treatment for DNA methylation detection.

### 2.7 Bisulfite sequencing PCR (BSP) method for detecting the methylation level of MGMT and hMLH1

The BSP method was used as previously described[25]. The primers for MGMT (200 bp) and hMLH1 (303 bp) were F:GCGTTTTTTTGTTTTTTTTAGGT R: AACGACCCAAACACTCACCAAA; F TGAGGCGGCGATAGATTAGGTATAGG R: AATACGAAATATCCAACCAATAAAAAC, which is was longer than the HRM methods used in humans. The Epitect Bisulfite Conversion Kit (Qiwu, Shanghai, China) was used to deaminate cytosine to uracil but to not 5-methyl-cytosine. The fragment of the premotor areas of MGMT and hMLH1 was amplified with PCR. The PCR product was then cloned into plasmid vectors using pMD19-T-vector (Takara) following the manufacturer’s instructions. Then, plasmid DNAs were transferred to competent DH5a cells cultured in L-agar plate culture medium. Finally, 10 signal clones were collected and amplified using PCR for sequencing. The primer was 5-GAGCGGATAACAATTTCACACAGG, 5-CGCCAGGGTTTTCCCAGTCACGAC and was sequenced by Sangon Biotech in Shanghai, China.

### 2.8 Statistical analysis

The SAS statistical package (Version 9.1) was used for data analyses. The significance level (alpha) was set at 5% for all analyses. The data were examined using a normality test, and linear regression analysis was used to determine the differences between the exposed workers and controls after adjusting for gender, smoking, drinking and age. Paired-samples T tests were used to compare the data from the year 2009 and with the data from 2013. Poisson regression was used to estimate the effects of BZ exposure and lifestyles on MN frequency.

## 3. Results

### 3.1 Benzene exposure assessment

Benzene concentrations were measured at all of the worksites of the painting workshop in an automobile factory. The painting workshop has improved its production techniques by switching from benzene-containing paints since 2009. The air benzene concentrations at 10 worksites were measured in the years 2010 and 2013. The range of benzene concentrations for an 8-h TWA was from 0 to 3.4 mg/m^3^ in 2010, and the mean/median air benzene was 1.12/0.8 mg/m^3^, while the benzene concentration in 2013 was less than 0.6 mg/m^3^. In 2010, approximately 25.7% (506 out of the 1969) of the benzene-exposed workers’ white blood cell counts were less than (4.5×10^9^)/L; while in 2013, only 9.3% (142 out of the 1520) benzene-exposed workers’ white blood cell counts were was less than (4.5×10^9^)/L. The benzene concentrations measured in the shoe factory in Wenzhou ranged from 2.6 mg/m^3^ to 57.0 mg/m^3^ (median, 6.4 mg/m^3^) in the air. This results were described in detail in a previous publication [26].

### 3.2 BZ exposure and its correlation with MN frequency, hemograms, and DNA methylation

The Poisson regression showed that the MN frequency in both of the exposed groups was significantly higher than that of the control group (*p*<0.01), and linear regression found that the WBC of the exposed groups was significantly lower than that of the control group (*p* = 0.01; Table 2).

**Table 2:** Markers of effects and methylation measured in benzene-exposed workers and controls.

Markers	Controls		Car factory workers (2013)		*P*^*a*^	Shoes factory workers		*P*^*a*^
	N	$\bar{X}$±SD	N	$\bar{X}$±SD		N	$\bar{X}$±SD	
White blood cell(×10^9^/L)	48	6.22±1.16	58	5.12±2.44	0.010	83	5.21±1.27	0.010
Red blood cell (×10^12^/L)	48	5.29±0.49	58	5.25±0.46	0.848	83	4.64±0.64	<0.001
Hemoglobin (g/L)	48	154.35±19.35	58	151.62±12.35	0.615	83	153.22±28.73	0.996
Platelet (×10^9^/L)	48	221.35±42.08	58	188.74±46.79	<0.001	83	185.73±42.47	<0.001
MN frequency(‰)	48	2.73±2.28	58	3.22±1.78	<0.001	83	3.67±1.92	<0.001
LINE1 (%)	48	54.09±5.84	58	51.53±7.39	0.072	83	45.90±7.76	<0.001
MGMT (%)	48	4.49±3.67	58	6.07±1.61	0.010	83	7.25±2.39	<0.001
hMLH1 (%)	48	10.61±4.84	58	9.47±5.45	0.754	83	14.26±8.74	0.010

The data were derived from General linear regression after adjusting for gender, age, smoking, and drinking, except MN frequency were analyzed by Poisson regression.

^a^ p value got from the compared with control with two benzene exposed groups separately.

The suitability of methylation specific (MS)-PCR for measuring biologically significant levels of LINE-1, MGMT, and hMLH1 methylation was assessed using HRM PCR (Table 2). The average percentage of methylated cytosines in LINE-1 sequences was (51.53±7.39)% for the car painting workers and (45.90±7.76)% for the shoe workers, which was significantly less than that of the control group (54.09±5.84; *p*<0.05). MGMT methylation showed a significant increase in the car painting workers (6.09±1.63)% and the shoe workers (7.23±2.30)% compared with the controls (4.54±3.32%; *p*<0.05). hMLH1 methylation showed a significant increase in the shoe workers (13.58±8.04)% compared with the controls (10.61±4.84%; *p*<0.05).

### 3.3 Follow-up of peripheral blood hemogram, chromosomal damage, and DNA methylation in the subcohort

To explore the progression of hematotoxicity and chromosomal damage over time, we compared the peripheral blood hemograms and MN frequencies in 2009 and 2013 for 35 benzene-exposed workers in the follow-up subcohort. Table 3 indicates the change in the peripheral blood hemograms and MN frequencies before and after follow-up. The mean white blood cell (mean±SD; 5.39±2.75 vs 4.62±1.44 (×10^9^/L), *p* = 0.109), red blood cell (mean±SD; 5.22±0.37 vs 4.04±0.41 (×10^12^/L), *p*<0.01), and hemoglobin (mean±SD; 151.43±12.12 vs 135.94±14.53 (g/L), *p*<0.01) counts from 2013 were significantly higher than those from 2009. Poisson regression also showed that the in 2013, the subjects had higher mean MN frequencies (3.51±1.88 (‰)) than in 2009 (2.86±2.07 (‰)), although the difference was not statistically significant.

**Table 3:** The change of peripheral blood biomarkers before and after follow-up.

Group	Number	$\bar{X}$±SD		*t*	*P*	*95%C I*^*a*^
		2009 year	2013 year			
White blood cell(×10^9^/L)	35	4.62±1.44	5.39±2.75	1.648	0.109	0.77 (-0.18–1.72)
Red blood cell (×10^12^/L)	35	4.04±0.41	5.22±0.37	17.178	<0.001	1.18(1.04–1.32)
Hemoglobin (g/L)	35	135.94±14.53	151.43±12.12	6.973	<0.001	15.49(10.97–20.00)
Platelet (×10^9^/L)	35	176.60±41.33	188.77±54.25	1.232	0.226	12.17(-7.91–32.25)
MN frequency(‰) ^a^	35	2.86±2.07	3.51±1.88	2.36	0.124	1.23(0.95–1.60)
LINE1	34	45.08±9.50	51.31±7.63	3.089	0.004	1.81 (1.90–9.31)
MGMT	34	6.31±3.78	6.66±1.80	0.609	0.547	0.47 (-1.11–2.06)
hMLH1	34	11.30±5.30	9.28±4.32	-2.096	0.045	-2.39(-4.73–0.06)

Data derived from Paired-samples T text; a 95%CI was the 95%Confidence interval of the difference.

^a^ MN frequency were derived from Poisson regression.

We converted WBC count and MN frequency to a dichotomous indicator of normal or reduced WBC count. We used the 4.50×10^9^/L as the cut-off point below which a worker’s WBC count was considered below normal. Because this index (4.50×10^9^/L) was used in the benzene-exposed workers’ health examination, when a worker’s WBC was less than 4.50×10^9^/L, he or she was placed on an observation list. We used the 5th percentile of MN frequency for the control workers as the cut-off point below which a worker’s MN frequency (4‰) was considered above normal, according to our previously study[27]. Thus, the WBC count and MN frequency were converted to dichotomous indicators of normal or reduced WBC count. Table 4 shows that the number of participants with reduced WBC in 2013 was significantly less than in 2009; Table 5 shows that although the number of participants with elevated MN frequency in 2013 was higher than in 2009, the difference was not statistically significant.

**Table 4:** The difference of reduced WBC between the year of 2009 and 2013.

2009 year	2013 year		Total(%)	*χ*^2^	*P*
	+	-			
+	14 (40)	11 (31)	25 (71)		
-	0 (0)	10 (29)	10 (29)		
Total(%)	14 (40)	21 (60)	35	9.027	0.002

Reduced WBC was defined as white blood cell count ≤ 4.5×10^9^/L.

+: number of workers with reduced WBC counts; -: number of workers with normal WBC counts; %: percentage of workers with reduced WBC counts.

**Table 5:** The difference of elevated MN frequency between the year of 2009 and 2013.

2009 year	2013 year		Total(%)	*χ*^2^	*p*
	+	-			
+	6 (17)	3 (9)	9 (26)		
-	12 (34)	14 (40)	26 (74)		
Total(%)	18 (51)	17 (49)	35	1.145	0.443

Micronucleus damage was defined as micronucleus frequency ≥ 4‰

+: number of workers with elevated MN frequency; -: number of workers with normal MN frequency; %: percentage of the workers with elevated MN frequency.

To explore the progression of DNA methylation over time, we compared the peripheral blood hemograms and MN frequencies between 2009 and 2013 in the follow-up subcohort. The degree of LINE-1 methylation in 2013 was significantly higher than it was in 2009 (2013 vs 2009; 51.31±7.63% vs 45.08±9.50%; p<0.01). Furthermore, the promoter methylation in hMLH1 in 2013 was less than in 2009 (2013 vs 2009; 11.30±5.30% vs 9.28±4.32%; *p*<0.05; Table 3).

### 3.4 In vitro results for promoter hypermethylation of hMLH1 after BQ treatment

**Fig 2:**
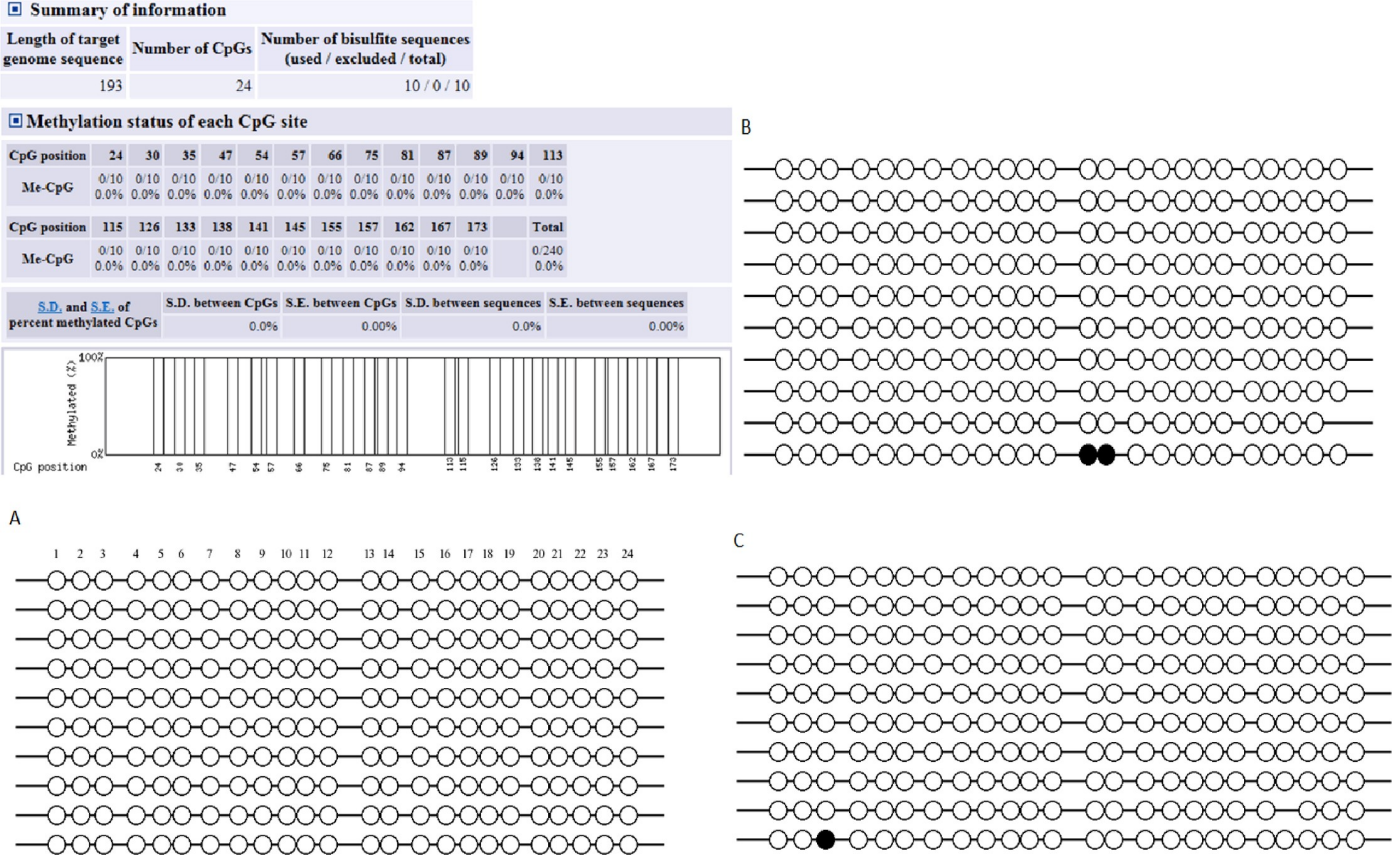
Methylation status of the CpG island in the MGMT promoter. The 24 CpG island were detected in MGMT promoter by BSP. (A) showed the control group, (B) indicated the AML-5 after treatment of benzoquinone for 72 hours, (C) indicated the AML-5 after treatment of hydroquinone for 72 hours.

**Fig 3:**
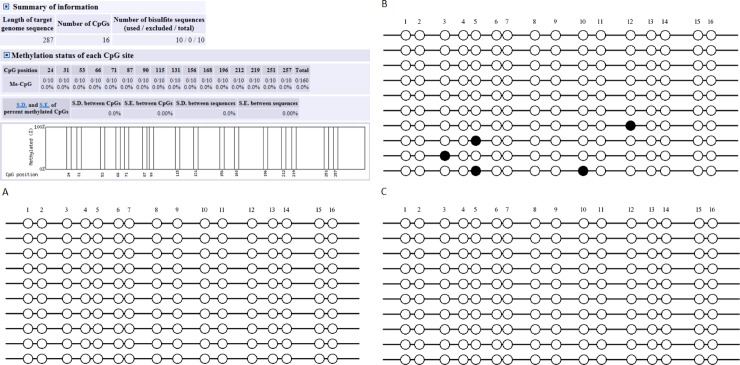
Methylation status of the CpG island in the MLH1 promoter. The 16 CpG island were detected in MLH1 promoter by BSP. (A) showed the control group, (B) indicated the hypemethylation of promoter region in MLH1 in AML-5 after treatment of benzoquinone for 72 hours, (C) indicated the AML-5 after treatment of hydroquinone for 72 hours.

An AML-5 cell line was treated with BQ and HQ to obtain additional 5’ sequences of MGMT and hMLH1 and better characterize the full extent of the MGMT and hMLH1. Twenty-four CpG islands in the MGMT promoter region and 16 CpG islands in the hMLH1 promoter region were detected. Fig 2 and S3 Table showed that there was no change in MGMT after BQ and HQ treatment. Fig 3 and S4 Table showed that after BQ treatment, several CpG islands in the promoter region of hMLH1 showed altered hypermethylation in the promoter region.

## 4. Discussion

The carcinogenic and hematotoxic effects of benzene are well recognized by the scientific community. Significant decreases in WBC, RBC and platelet counts and increases in MN frequency have been observed in human populations exposed to relatively high or low levels of benzene[26, 28, 29]. We showed that workers exposed to very low levels of benzene have elevated levels of micronucleus (MN) induction and decreased peripheral hemograms compared with unexposed controls, which is consistent with a previous report [28, 30, 31]. To explore the progression of chromosomal damage and hematotoxicity in Chinese workers exposed to benzene, the CBMN test and hemograms were performed prospectively in the peripheral blood of 35 benzene-exposed workers in both 2009 and 2013. The benzene concentration in air of the worksites in 2013 was significantly lower than in 2009 as a result of improved processes and changes in the coating used. The hematotoxic effects in exposed workers were significantly improved in 2013 compared with 2009 as a result of the reduction in benzene exposure, but the chromosomal damage indicated by the MN frequency was worse in 2013 than in 2009.

Epidemiological evidence indicates that exposure to benzene is associated with hematotoxicity and is involved in the development of aplastic anemia and leukemia, even in workers exposed to less than 1 ppm of benzene in air [32, 33]. Our results indicated that the MN frequency in the benzene-exposed workers was higher in 2013 than in 2009. This implies that chromosomal damage is still serious even when the benzene concentration in the air was less than 0.6 mg/m3. The formation of MN is the result of chromosome breakage and loss caused by unrepaired or misrepaired DNA lesions or chromosome malsegregation resulting from mitotic malfunction[34]. Recent studies found that DNA rearrangements and mutations in MN could be incorporated into the genome of a developing cancer cell [35]. We can deduce that ever-benzene-exposed workers would have an increased chance of developing cancer in the future, even if they had not been exposed to benzene for many years. This was consistent with a cohort meta-analysis [36] that found evidence of an association between occupational benzene exposure and multiple myeloma (MM), acute lymphocytic leukemia (ALL), and chronic lymphocytic leukemia (CLL). The study [37, 38] showed that the standardized mortality ratios (SMR) for leukemia were 1.09 for 1 ppm-year or lower, 3.22 for 40–100 ppm-year, 11.86 for 200–399 ppm-year and 66.37 for 400 ppm-year and higher in the cohort study. [39] reported that in five cases of multiple myeloma in exposed workers in a cohort, four cases were exposed to the lowest exposure category defined in 2002 (1 ppm-day to 30.99 ppm-years of exposure). Furthermore, Glass and colleagues[32] performed a nested case-control study and found that the risk of leukemia was increased at cumulative exposures above 2 ppm-years and with intensity of exposure of more than 0.8 ppm for the highest-exposure jobs.

Both in vitro and in vivo studies have shown that carcinogenic agents induce DNA methylation changes in normal tissues similar to those found in malignant cells [40, 41]. In our study, we observed a benzene-related decrease in the methylation of LINE-1 repeated elements and elevated MGMT and hMLH1 promoters in benzene-exposed workers. Our in vitro study found hypermethylation in the promoter region of hMLH1 after BQ treatment in AML-5 cells. In our exploration of the progression of DNA methylation in workers exposed to benzene, we found an increase in the methylation of LINE-1 and a decrease in hHMLH1 in 2013 compared with 2009. We did not find a direct correlation between the levels of LINE-1, MGMT, and hMLH1 methylation in all subjects or in the benzene-exposed workers, indicating that the alteration of global methylation and gene-specific methylation occurred independently. This finding is consistent with a previous report [9].

Global genomic hypomethylation is a common occurrence in cancer tissues that are frequently exposed to environmental carcinogens because of the heavy methylation of repetitive elements, which are easier to detect than previous methods that quantitated the total genomic 5-methylcytosine. Numerous studies have quantitated DNA methylation in long interspersed nuclear element-1 (LINE-1) repetitive elements as a surrogate of genome-wide methylation [14, 40]. Our observation is consistent with the global hypomethylation frequently observed in other populations, such as petrochemical workers[42], gas station attendants and traffic police officers [40, 44], that are exposed to low levels of benzene. Additionally, our finders were confirmed in an in vitro study. Hu et al. reported [43] that HQ and BQ could induce global DNA hypomethylation at levels that differed significantly from controls (p < 0.05). Ji et al. [41] found that HQ can induce global DNA hypomethylation in human TK6 lymphoblastoid cells. However, still a study only found a week association between hypomethylation and benzene exposure[44], we think this perhaps the exposure dose in this study was much lower or the different selected subjects.

Because of its high prognostic and predictive relevance, assessment of the MGMT and hMLH1status as a prognosticator has become state-of-the-art in current and planned clinical trials of cancer [10, 45, 46]. Our results show an exposure-related increase in the methylation of MGMT and hMLH1. Previous report indicated MGMT methylation was negatively associated with the benzene exposure and the degree of DNA damage[47]. The in vitro particularly validated the occurrence of hMLH1 hypermethylation after BQ treatment. Numerous studies have reported the elevated promoter methylation in cancer tissues, but to the best of our knowledge, none has reported the methylation status of two genes in benzene-exposed workers. Psofaki et al. [45] found that MGMT presented a statistically significant increase in promoter methylation between the less- and more-tumorigenic forms of colorectal adenomas (tubular vs tubullovillous and villous adenomas), and Wu et al. [48] previously observed in our lab that MGMT promoter methylation was elevated in vinyl chloride monomer-induced chromosome-damaged subjects. However, Duan et al. [9] observed a PAH-related decrease in the methylation of the MGMT promoter, although their in vitro test found that MGMT was methylated in low-dose (2.5 lg/ml) coke oven emissions exposure groups and demethylated in high-dose groups.

MS-HRM is a useful and very sensitive method for detecting methylation in an unmethylated background to rapidly assess the presence of DNA methylation [10, 12]. It is a simple method for simultaneously assessing the DNA methylation of several loci in the genome; furthermore, it is an in-tube method, meaning that is important for diagnostic laboratories not only because of its rapidity but because it eliminates the possibility of PCR product contamination[10]. Additionally, it can semi-quantitatively estimate the amount of homogeneous methylation. It is requested as a prognostic tool in the routine diagnosis of cancer or environmental toxin exposure, and because of its high prognostic and predictive relevance, assessment of the LINE-1 status has become a state-of-the-art prognostic tool for use with current and future benzene-exposed workers. Our purpose was to find a useful and rapid method for detecting benzene exposure. However, MS-HRM cannot estimate the amount of heterogeneous methylation caused by the formation of heteroduplexes between the different epialleles in heterogeneously methylated samples[49].

In conclusion, we have demonstrated in this study that the persistence of residual genotoxic damage induced by benzene can be characterized in MN frequencies, and the progression of chromosomal damage was evident at the end of a 4-year follow-up, even when the benzene exposure was significantly reduced. Furthermore, the follow-up and in vitro examination data showed that low-level benzene exposure is associated with global DNA hypomethylation and DNA repair gene-specific hypermethylation (hMLH1) in normal subjects and that the DNA methylation patterns changed with the reduction of benzene exposure. Further studies are required to better define these findings and examine additional gene-specific methylation modifications in larger cohorts.

## Supporting information

S1 Table The demographic characteristics, peripheral blood, micronuclear frequency and DNA methylation of the study subjects. (DOCX)

S2 Table The DNA methylation, peripheral blood, and micronuclear frequency of the follow-up painting workers. (DOCX)

S3 Table The premotor methylation of MGMT in AML-5 cells by BSP methods. S3a The premotor methylation of MGMT after the treatment of benzoquinone of AML-5 cells by BSP methods; S3b The premotor methylation of MGMT after the treatment of hydroquinone of AML-5 cells by BSP methods.
(DOCX)

S4 Table The premotor methylation of MLH1 in AML-5 cells by BSP methods. S4a The premotor methylation of MLH1 after the treatment of benzoquinone of AML-5 cells by BSP methods; S4b The premotor methylation of MLH1 after the treatment of hydroquinone of AML-5 cells by BSP methods.
(DOCX)
