# Supplementary material for: The prevalence and persistence of aberrant promoter DNA methylation in benzene-exposed Chinese workers
Source: PLoS One. 2019 Aug 5;14(8):e0220500. doi: 10.1371/journal.pone.0220500 (PMC6681966; doi:10.1371/journal.pone.0220500)
Supplement: S1 Table — (DOCX) [file pone.0220500.s001.docx]

Table S1 The demographic characteristics, peripheral blood, micronuclear frequency and DNA methylation of the study subjects.

| group | number | gender | age | smoking | drinking | WBC(×10^9^) | RBC(×10^9^) | HB(g/L) | Platelet(×10^9^) | MN(‰) | LINE1(%) | MGMT(%) | MLH1(%) |
| --- | --- | --- | --- | --- | --- | --- | --- | --- | --- | --- | --- | --- | --- |
| 1 | 1 | 1 | 24 | 0 | 1 | 2.9 | 4.89 | 142 | 142 | 3 | 69.77 | 5.05 | 6.52 |
| 1 | 2 | 1 | 25 | 1 | 0 | 3.1 | 5.3 | 152 | 170 | 6 | 68.9 | 5.96 | 6.78 |
| 1 | 3 | 1 | 21 | 0 | 0 | 3.1 | 5.31 | 157 | 182 | 2 | 79.72 | 6.21 | 21.66 |
| 1 | 4 | 1 | 24 | 1 | 0 | 3.2 | 5.4 | 152 | 153 | 5 | 74.99 | 4.22 | 6.51 |
| 1 | 5 | 1 | 26 | 1 | 1 | 3.3 | 4.89 | 139 | 123 | 2 | 69.86 | 4.42 | 4.57 |
| 1 | 6 | 0 | 26 | 1 | 1 | 3.3 | 4.64 | 135 | 142 | 5 | 87.96 | 5.16 | 4.92 |
| 1 | 7 | 1 | 36 | 1 | 1 | 3.3 | 5.14 | 150 | 179 | 5 | 67.61 | 5.74 | 5.46 |
| 1 | 8 | 1 | 25 | 1 | 1 | 3.4 | 5.33 | 153 | 197 | 0 | 76.89 | 4.46 | 6.84 |
| 1 | 9 | 0 | 24 | 1 | 1 | 3.5 | 4.79 | 135 | 104 | 2 | 67.15 | 6.16 | 7.92 |
| 1 | 10 | 1 | 22 | 0 | 0 | 3.5 | 4.59 | 137 | 145 | 5 | 81.48 | 4.89 | 5.51 |
| 1 | 11 | 1 | 44 | 0 | 0 | 3.5 | 5.2 | 157 | 198 | 4 | 78.3 | 5.18 | 6.04 |
| 1 | 12 | 1 | 44 | 0 | 0 | 3.5 | 5.2 | 157 | 198 | 4 | 78.3 | 5.18 | 6.04 |
| 1 | 13 | 1 | 21 | 1 | 0 | 3.6 | 5.75 | 158 | 170 | 4 | 73.18 | 4.91 | 5.35 |
| 1 | 14 | 0 | 22 | 1 | 1 | 3.6 | 4.37 | 133 | 210 | 1 | 68.16 | 4.13 | 6.13 |
| 1 | 15 | 1 | 30 | 0 | 0 | 3.8 | 4.84 | 158 | 155 | 1 | 72.58 | 5.78 | 5.85 |
| 1 | 16 | 1 | 40 | 0 | 0 | 3.8 | 5.71 | 167 | 221 | 1 | 70.94 | 4.54 | 6.39 |
| 1 | 17 | 1 | 25 | 0 | 0 | 3.8 | 5.38 | 150 | 241 | 4 | 75.76 | 6.83 | 10.82 |
| 1 | 18 | 0 | 28 | 1 | 0 | 4 | 4.66 | 134 | 167 | 5 | 77.71 | 8.99 | 11.29 |
| 1 | 19 | 0 | 37 | 1 | 1 | 4 | 4.73 | 131 | 194 | 7 | 77.51 | 5.62 | 9.19 |
| 1 | 20 | 1 | 25 | 1 | 0 | 4.1 | 5.16 | 142 | 161 | 4 | 78.76 | 4.97 | 5.37 |
| 1 | 21 | 1 | 28 | 1 | 0 | 4.1 | 5.58 | 170 | 294 | 5 | 69.41 | 4.93 | 7.78 |
| 1 | 22 | 1 | 24 | 0 | 0 | 4.2 | 4.98 | 140 | 179 | 3 | 74.11 | 4.9 | 22.05 |
| 1 | 23 | 0 | 26 | 1 | 1 | 4.2 | 4.89 | 137 | 191 | 7 | 58.35 | 7.35 | 5.13 |
| 1 | 24 | 1 | 39 | 1 | 0 | 4.2 | 5.54 | 157 | 200 | 3 | 68.59 | 5.92 | 5.64 |
| 1 | 25 | 1 | 29 | 1 | 1 | 4.3 | 5.33 | 156 | 145 | 2 | 75.44 | 6.05 | 7.08 |
| 1 | 26 | 1 | 23 | 0 | 0 | 4.3 | 5.13 | 145 | 218 | 4 | 72.93 | 5.17 | 6.64 |
| 1 | 27 | 1 | 31 | 1 | 0 | 4.4 | 5.16 | 148 | 195 | 2 | 75.7 | 5 | 23.7 |
| 1 | 28 | 1 | 32 | 1 | 0 | 4.5 | 5.06 | 151 | 170 | 2 | 79.42 | 8.09 | 11.35 |
| 1 | 29 | 1 | 34 | 1 | 0 | 4.5 | 5.33 | 162 | 218 | 4 | 85.85 | 7.09 | 9.63 |
| 1 | 30 | 1 | 31 | 0 | 0 | 4.6 | 5.37 | 157 | 163 | 4 | 77.14 | 10.15 | 6.21 |
| 1 | 31 | 1 | 31 | 1 | 0 | 4.8 | 5.76 | 163 | 125 | 6 | 78.19 | 7.71 | 10.53 |
| 1 | 32 | 1 | 33 | 0 | 0 | 4.8 | 5.16 | 158 | 146 | 2 | 56.12 | 6.85 | 10.29 |
| 1 | 33 | 1 | 30 | 1 | 0 | 4.8 | 4.91 | 145 | 149 | 3 | 81.4 | 6.71 | 7.49 |
| 1 | 34 | 1 | 29 | 1 | 1 | 4.8 | 5.48 | 170 | 168 | 1 | 63 | 6.72 | 6.19 |
| 1 | 35 | 1 | 41 | 0 | 0 | 4.8 | 5.57 | 159 | 237 | 2 | 72.85 | 6.23 | 9.16 |
| 1 | 36 | 0 | 22 | 1 | 1 | 4.9 | 4.61 | 140 | 191 | 2 | 70.98 | 4.76 | 6.04 |
| 1 | 37 | 1 | 43 | 1 | 1 | 5 | 4.52 | 139 | 80 | 4 | 76.01 | 8.88 | 11.85 |
| 1 | 38 | 1 | 21 | 1 | 1 | 5 | 5.68 | 165 | 173 | 2 | 74.77 | 6.59 | 6.32 |
| 1 | 39 | 1 | 27 | 0 | 0 | 5 | 5.27 | 157 | 199 | 3 | 88.62 | 6.72 | 13.47 |
| 1 | 40 | 1 | 26 | 1 | 0 | 5 | 4.84 | 142 | 255 | 4 | 77.43 | 4.28 | 6.3 |
| 1 | 41 | 1 | 22 | 1 | 1 | 5.1 | 5.51 | 161 | 151 | 0 | 85.71 | 8.19 | 9.59 |
| 1 | 42 | 1 | 31 | 1 | 0 | 5.1 | 5.35 | 163 | 154 | 1 | 83.33 | 11.86 | 12.87 |
| 1 | 43 | 1 | 29 | 0 | 0 | 5.1 | 5.19 | 157 | 195 | 1 | 78.59 | 4.77 | 6.36 |
| 1 | 44 | 1 | 23 | 0 | 0 | 5.3 | 5.02 | 145 | 183 | 1 | 68.75 | 5.69 | 24.99 |
| 1 | 45 | 1 | 23 | 0 | 0 | 5.3 | 5.02 | 145 | 183 | 1 | 68.75 | 5.69 | 24.99 |
| 1 | 46 | 1 | 21 | 1 | 0 | 5.5 | 5.57 | 166 | 182 | 4 | 84.24 | 8.16 | 8.7 |
| 1 | 47 | 1 | 35 | 1 | 1 | 5.6 | 5.16 | 147 | 187 | 4 | 67.54 | 4.07 | 4.67 |
| 1 | 48 | 1 | 23 | 0 | 1 | 5.7 | 5.89 | 175 | 167 | 1 | 70.59 | 4.85 | 6.13 |
| 1 | 49 | 1 | 32 | 1 | 0 | 5.9 | 5.02 | 142 | 292 | 4 | 77.4 | 7.1 | 6.62 |
| 1 | 50 | 1 | 24 | 1 | 0 | 6.5 | 5.41 | 159 | 230 | 3 | 88.07 | 5.39 | 5.35 |
| 1 | 51 | 1 | 21 | 0 | 0 | 6.8 | 5.35 | 171 | 255 | 3 | 68.99 | 5.97 | 5.89 |
| 1 | 52 | 1 | 43 | 0 | 0 | 7.1 | 6 | 122 | 130 | 1 | 72.38 | 5.08 | 5.87 |
| 1 | 53 | 1 | 30 | 1 | 0 | 8.1 | 6 | 168 | 223 | 6 | 88.15 | 2.92 | 20.19 |
| 1 | 54 | 0 | 23 | 1 | 1 | 8.1 | 4.79 | 137 | 279 | 3 | 69.99 | 6.84 | 18.72 |
| 1 | 55 | 1 | 28 | 1 | 0 | 9.6 | 5.69 | 172 | 198 | 3 | 73.63 | 6.09 | 6.77 |
| 1 | 56 | 1 | 30 | 0 | 0 | 9.6 | 7.14 | 136 | 216 | 4 | 75.91 | 5.28 | 14.16 |
| 1 | 57 | 1 | 44 | 0 | 0 | 11.7 | 4.97 | 163 | 253 | 6 | 81.86 | 6.96 | 13.06 |
| 1 | 58 | 1 | 31 | 0 | 1 | 18.1 | 5.53 | 165 | 321 | 6 | 76.76 | 8.57 | 12.56 |
| 2 | 59 | 0 | 30 | 1 | 0 | 3 | 4.04 | 210 | 175 | 5 | 74.7 | 6.42 | 22.32 |
| 2 | 60 | 1 | 30 | 1 | 0 | 3.7 | 4.9 | 130 | 134 | 2 | 82.93 | 6.45 | 8.74 |
| 2 | 61 | 1 | 40 | 1 | 1 | 3.7 | 5.92 | 168 | 238 | 4 | 74.9 | 6.54 | 10.77 |
| 2 | 62 | 1 | 51 | 1 | 1 | 3.7 | 5.92 | 136 | 238 | 3 | 74.9 | 6.89 | 17.68 |
| 2 | 63 | 0 | 37 | 1 | 1 | 3.8 | 4.8 | 176 | 176 | 6 | 70.8 | 7.08 | 37.27 |
| 2 | 64 | 0 | 47 | 1 | 1 | 3.8 | 4.92 | 93 | 181 | 4 | 74.3 | 4.97 | 19.53 |
| 2 | 65 | 0 | 30 | 1 | 1 | 3.8 | 4.17 | 146 | 181 | 2 | 79.2 | 6.3 | 19.75 |
| 2 | 66 | 0 | 48 | 1 | 1 | 3.9 | 4.45 | 146 | 145 | 3 | 75.7 | 7.46 | 14.9 |
| 2 | 67 | 0 | 40 | 1 | 1 | 4 | 4.63 | 120 | 150 | 4 | 71.64 | 7.71 | 41.09 |
| 2 | 68 | 0 | 32 | 1 | 1 | 4 | 3.49 | 150 | 145 | 3 | 74.6 | 7.07 | 20.07 |
| 2 | 69 | 0 | 20 | 1 | 1 | 4 | 4.18 | 139 | 145 | 4 | 77.3 | 6.38 | 21.32 |
| 2 | 70 | 1 | 38 | 0 | 0 | 4 | 4.8 | 214 | 177 | 5 | 76 | 6.1 | 9.76 |
| 2 | 71 | 1 | 38 | 0 | 1 | 4 | 4.8 | 114 | 177 | 6 | 76 | 6.07 | 15.77 |
| 2 | 72 | 1 | 36 | 1 | 0 | 4 | 5.11 | 176 | 202 | 1 | 75.7 | 5.8 | 17.28 |
| 2 | 73 | 1 | 19 | 0 | 0 | 4.1 | 4.53 | 130 | 138 | 1 | 73.61 | 23.61 | 7.09 |
| 2 | 74 | 0 | 46 | 1 | 1 | 4.1 | 4.8 | 150 | 173 | 5 | 72.49 | 9.51 | 39.13 |
| 2 | 75 | 1 | 18 | 1 | 0 | 4.1 | 4.8 | 163 | 221 | 4 | 73.58 | 7.91 | 5.04 |
| 2 | 76 | 0 | 49 | 1 | 1 | 4.1 | 4.88 | 110 | 177 | 3 | 69.98 | 7.97 | 8.59 |
| 2 | 77 | 0 | 41 | 1 | 1 | 4.1 | 4.31 | 158 | 164 | 2 | 77.5 | 4.77 | 39.38 |
| 2 | 78 | 0 | 47 | 1 | 0 | 4.2 | 4.67 | 159 | 164 | 4 | 70.24 | 8.41 | 36.79 |
| 2 | 79 | 0 | 42 | 1 | 1 | 4.2 | 4.8 | 167 | 175 | 3 | 70.55 | 9.06 | 4.67 |
| 2 | 80 | 1 | 24 | 1 | 1 | 4.2 | 4.85 | 129 | 281 | 4 | 70.3 | 7.84 | 5.75 |
| 2 | 81 | 1 | 19 | 1 | 0 | 4.2 | 3.91 | 129 | 164 | 1 | 74.7 | 5.5 | 21.16 |
| 2 | 82 | 1 | 25 | 1 | 0 | 4.2 | 5.04 | 121 | 242 | 4 | 75.3 | 5.76 | 18.46 |
| 2 | 83 | 0 | 33 | 1 | 1 | 4.2 | 4.92 | 112 | 312 | 4 | 75.3 | 6.67 | 18.32 |
| 2 | 84 | 0 | 47 | 1 | 1 | 4.3 | 4.55 | 134 | 238 | 0 | 69.28 | 8.97 | 4.75 |
| 2 | 85 | 0 | 29 | 1 | 1 | 4.3 | 3.47 | 159 | 138 | 4 | 74.7 | 5.14 | 16.09 |
| 2 | 86 | 0 | 37 | 1 | 1 | 4.3 | 4.67 | 186 | 176 | 4 | 78.1 | 5.85 | 16.3 |
| 2 | 87 | 1 | 21 | 0 | 0 | 4.3 | 5.37 | 145 | 164 | 5 | 75.7 | 6.93 | 13.41 |
| 2 | 88 | 0 | 38 | 1 | 1 | 4.4 | 4.63 | 123 | 246 | 5 | 68.06 | 7.8 | 4.68 |
| 2 | 89 | 0 | 39 | 1 | 1 | 4.4 | 4.18 | 148 | 138 | 4 | 77.2 | 6.72 | 22.11 |
| 2 | 90 | 0 | 42 | 1 | 1 | 4.4 | 4.24 | 114 | 157 | 8 | 74.7 | 5.62 | 12.59 |
| 2 | 91 | 1 | 21 | 0 | 0 | 4.4 | 4.85 | 196 | 181 | 1 | 75.3 | 7.31 | 11.37 |
| 2 | 92 | 0 | 33 | 1 | 1 | 4.5 | 3.36 | 123 | 182 | 3 | 76.2 | 7.61 | 36.8 |
| 2 | 93 | 0 | 19 | 1 | 1 | 4.5 | 3.51 | 163 | 302 | 4 | 75.6 | 5.68 | 18.32 |
| 2 | 94 | 0 | 31 | 1 | 1 | 4.5 | 3.53 | 167 | 157 | 4 | 75.7 | 4.8 | 20.87 |
| 2 | 95 | 1 | 37 | 1 | 0 | 4.5 | 3.97 | 131 | 121 | 2 | 75.2 | 6.46 | 22.95 |
| 2 | 96 | 0 | 32 | 1 | 1 | 4.5 | 4.07 | 155 | 238 | 4 | 79 | 6.16 | 13.58 |
| 2 | 97 | 0 | 23 | 1 | 1 | 4.6 | 4.9 | 125 | 130 | 6 | 78.45 | 7.77 | 9.89 |
| 2 | 98 | 0 | 23 | 1 | 1 | 4.7 | 4.49 | 122 | 150 | 1 | 75.4 | 7.08 | 11.95 |
| 2 | 99 | 0 | 37 | 1 | 1 | 4.8 | 4.41 | 208 | 173 | 7 | 75.1 | 6.39 | 24.59 |
| 2 | 100 | 0 | 19 | 1 | 1 | 4.8 | 4.63 | 172 | 174 | 0 | 75.8 | 6.11 | 7.61 |
| 2 | 101 | 0 | 24 | 1 | 1 | 4.8 | 4.6 | 127 | 220 | 5 | 71.44 | 15.18 | 11.89 |
| 2 | 102 | 1 | 33 | 1 | 0 | 4.9 | 5.49 | 156 | 175 | 6 | 72 | 6.83 | 11.9 |
| 2 | 103 | 1 | 21 | 1 | 1 | 4.9 | 3.3 | 128 | 228 | 4 | 78.09 | 5.76 | 8.79 |
| 2 | 104 | 1 | 25 | 0 | 0 | 4.9 | 5.1 | 164 | 239 | 4 | 62.42 | 10.61 | 8.69 |
| 2 | 105 | 0 | 22 | 1 | 1 | 5 | 4.13 | 115 | 198 | 2 | 75.5 | 7.96 | 18.42 |
| 2 | 106 | 1 | 21 | 0 | 0 | 5.3 | 5.2 | 161 | 189 | 4 | 76.92 | 7.13 | 7.1 |
| 2 | 107 | 0 | 37 | 1 | 1 | 5.4 | 4.02 | 93 | 174 | 7 | 76.1 | 6.42 | 10.89 |
| 2 | 108 | 0 | 30 | 1 | 1 | 5.5 | 4.2 | 142 | 150 | 5 | 72.49 | 12.83 | 10.97 |
| 2 | 109 | 0 | 51 | 1 | 1 | 5.5 | 4 | 125 | 228 | 4 | 67.28 | 6.56 | 14.58 |
| 2 | 110 | 1 | 46 | 1 | 1 | 5.6 | 4.9 | 185 | 201 | 6 | 75.6 | 9.67 | 13.9 |
| 2 | 111 | 1 | 21 | 0 | 1 | 5.6 | 3.6 | 176 | 176 | 4 | 72.27 | 7.46 | 5.04 |
| 2 | 112 | 1 | 43 | 1 | 1 | 5.7 | 4.8 | 170 | 116 | 3 | 79.77 | 6.5 | 6.94 |
| 2 | 113 | 1 | 20 | 0 | 0 | 5.7 | 5.1 | 164 | 157 | 5 | 74.71 | 6.25 | 9.76 |
| 2 | 114 | 0 | 34 | 1 | 1 | 5.8 | 4.53 | 214 | 157 | 1 | 75.6 | 6.08 | 8.5 |
| 2 | 115 | 0 | 24 | 1 | 1 | 5.9 | 4.17 | 170 | 182 | 4 | 75 | 6.38 | 22.59 |
| 2 | 116 | 1 | 44 | 1 | 1 | 5.9 | 5.25 | 182 | 182 | 4 | 75 | 6.84 | 18.73 |
| 2 | 117 | 0 | 33 | 1 | 1 | 5.9 | 4 | 115 | 215 | 2 | 75.66 | 7.1 | 7.36 |
| 2 | 118 | 1 | 21 | 1 | 1 | 5.9 | 4.2 | 114 | 304 | 6 | 75.8 | 7.1 | 6.81 |
| 2 | 119 | 0 | 25 | 1 | 1 | 6 | 4.1 | 182 | 177 | 2 | 71.42 | 7.37 | 5.11 |
| 2 | 120 | 1 | 35 | 0 | 0 | 6.1 | 4.2 | 149 | 185 | 2 | 83.06 | 5.7 | 8.26 |
| 2 | 121 | 1 | 24 | 0 | 0 | 6.2 | 5.3 | 185 | 210 | 9 | 72.25 | 8.58 | 8.11 |
| 2 | 122 | 1 | 19 | 0 | 0 | 6.3 | 4.44 | 144 | 138 | 1 | 75.6 | 6.82 | 14.27 |
| 2 | 123 | 0 | 18 | 1 | 1 | 6.3 | 4.6 | 151 | 173 | 6 | 75.1 | 6.21 | 5.66 |
| 2 | 124 | 1 | 26 | 0 | 0 | 6.3 | 5.4 | 150 | 174 | 0 | 76.65 | 7.58 | 5.74 |
| 2 | 125 | 0 | 19 | 1 | 1 | 6.3 | 4.1 | 121 | 252 | 2 | 68.72 | 7.65 | 5.59 |
| 2 | 126 | 0 | 19 | 1 | 1 | 6.6 | 4.8 | 213 | 213 | 3 | 86.22 | 7.34 | 6.57 |
| 2 | 127 | 1 | 25 | 1 | 0 | 6.6 | 5.76 | 147 | 261 | 7 | 75.2 | 7.24 | 9.05 |
| 2 | 128 | 0 | 29 | 1 | 0 | 6.7 | 4.63 | 187 | 175 | 5 | 75.5 | 7.44 | 17.47 |
| 2 | 129 | 1 | 37 | 1 | 1 | 6.8 | 4.55 | 212 | 164 | 5 | 76.5 | 7.68 | 15.51 |
| 2 | 130 | 1 | 33 | 1 | 1 | 6.8 | 4.9 | 153 | 181 | 4 | 72.79 | 6.51 | 11.01 |
| 2 | 131 | 1 | 22 | 0 | 0 | 6.8 | 5.2 | 194 | 194 | 0 | 69.85 | 8.5 | 4.66 |
| 2 | 132 | 1 | 21 | 1 | 1 | 7 | 5.3 | 177 | 177 | 2 | 76.96 | 7.31 | 6.51 |
| 2 | 133 | 0 | 26 | 1 | 0 | 7.1 | 4.4 | 134 | 138 | 2 | 77.51 | 7.28 | 7.95 |
| 2 | 134 | 1 | 20 | 0 | 0 | 7.2 | 4.88 | 175 | 182 | 4 | 77.4 | 5.63 | 22.4 |
| 2 | 135 | 1 | 30 | 0 | 0 | 7.2 | 5 | 162 | 207 | 5 | 77.48 | 6.25 | 7.71 |
| 2 | 136 | 1 | 28 | 1 | 0 | 7.3 | 5.5 | 172 | 177 | 2 | 81.29 | 6.29 | 19.94 |
| 2 | 137 | 1 | 30 | 0 | 0 | 7.4 | 7.1 | 167 | 150 | 6 | 88 | 5.93 | 10.98 |
| 2 | 138 | 0 | 20 | 1 | 1 | 7.4 | 4.2 | 136 | 236 | 1 | 72.2 | 6.73 | 7.25 |
| 2 | 139 | 1 | 25 | 1 | 0 | 7.5 | 5.1 | 166 | 103 | 2 | 70.96 | 6.38 | 8.54 |
| 2 | 140 | 0 | 47 | 1 | 1 | 7.5 | 5 | 162 | 145 | 6 | 80.06 | 5.62 | 11.53 |
| 2 | 141 | 1 | 24 | 1 | 1 | 9.5 | 5.2 | 160 | 173 | 4 | 76.8 | 6.34 | 12.53 |
| 3 | 142 | 1 | 61 | 0 | 0 | 6.12 | 6.21 | 225 | 173 | 3 | 77.83 | 2.33 | 6.09 |
| 3 | 143 | 0 | 58 | 1 | 0 | 5 | 5.1 | 144 | 174 | 1 | 77.32 | 2.71 | 10.69 |
| 3 | 144 | 1 | 59 | 1 | 1 | 5.32 | 5.2 | 147 | 220 | 1 | 78.49 | 2.27 | 7.88 |
| 3 | 145 | 0 | 63 | 1 | 0 | 10 | 5.6 | 172 | 175 | 1 | 77.69 | 2.01 | 6.12 |
| 3 | 146 | 0 | 69 | 1 | 1 | 6.3 | 5 | 153 | 228 | 9 | 76.58 | 2.99 | 7.84 |
| 3 | 147 | 0 | 58 | 1 | 1 | 6.3 | 5.2 | 174 | 239 | 3 | 77.33 | 19.15 | 16.01 |
| 3 | 148 | 0 | 40 | 1 | 1 | 6.8 | 5.4 | 175 | 198 | 2 | 78.52 | 2.86 | 19.73 |
| 3 | 149 | 1 | 75 | 1 | 0 | 7.2 | 5.5 | 134 | 189 | 6 | 78.81 | 1.64 | 17.07 |
| 3 | 150 | 0 | 62 | 1 | 0 | 7.5 | 5.4 | 175 | 220 | 5 | 77.83 | 1.78 | 6.6 |
| 3 | 151 | 1 | 41 | 1 | 0 | 6.1 | 5.89 | 162 | 175 | 1 | 67.05 | 3.51 | 8.23 |
| 3 | 152 | 1 | 42 | 1 | 0 | 5.12 | 5.89 | 152 | 208 | 3 | 69.51 | 3.47 | 7.97 |
| 3 | 153 | 0 | 33 | 1 | 0 | 6.1 | 5.5 | 167 | 239 | 1 | 80.3 | 5.68 | 6.57 |
| 3 | 154 | 0 | 71 | 1 | 1 | 5.5 | 6.7 | 154 | 267 | 4 | 79 | 2.86 | 15.88 |
| 3 | 155 | 0 | 47 | 1 | 0 | 6.3 | 6.2 | 122 | 250 | 2 | 83.12 | 3.32 | 7.53 |
| 3 | 156 | 1 | 33 | 0 | 0 | 6.3 | 5.1 | 154 | 241 | 0 | 80.51 | 3.41 | 16.52 |
| 3 | 157 | 1 | 67 | 1 | 1 | 6.8 | 6.03 | 153 | 187 | 4 | 77.35 | 1.84 | 5.5 |
| 3 | 158 | 1 | 79 | 1 | 0 | 7.2 | 5.2 | 169 | 210 | 5 | 77.21 | 4.04 | 21.08 |
| 3 | 159 | 0 | 72 | 1 | 1 | 7.5 | 5.1 | 161 | 201 | 3 | 76.65 | 3 | 17.63 |
| 3 | 160 | 1 | 25 | 1 | 0 | 9 | 5.2 | 157 | 180 | 1 | 76.68 | 3.15 | 7.36 |
| 3 | 161 | 1 | 25 | 1 | 0 | 8.4 | 4.4 | 153 | 289 | 0 | 73.43 | 3.53 | 8.43 |
| 3 | 162 | 1 | 29 | 1 | 0 | 5 | 5 | 194 | 249 | 2 | 56.84 | 11.3 | 7.27 |
| 3 | 163 | 1 | 29 |  |  | 5.68 | 5.2 | 127 | 301 | 0 | 80.72 | 3.6 | 5.94 |
| 3 | 164 | 0 | 38 | 1 | 1 | 5 | 5.4 | 134 | 170 | 1 | 78.51 | 3.73 | 11.67 |
| 3 | 165 | 1 | 58 | 0 | 0 | 5.5 | 5.5 | 175 | 170 | 2 | 75.5 | 4.33 | 6.66 |
| 3 | 166 | 0 | 63 |  | 1 | 6.3 | 5.5 | 162 | 269 | 5 | 80.51 | 3.18 | 6.1 |
| 3 | 167 | 1 | 60 | 1 | 0 | 6.3 | 4.88 | 172 | 297 | 2 | 78.51 | 3.93 | 7.26 |
| 3 | 168 | 0 | 65 | 1 | 1 | 5.6 | 5 | 167 | 315 | 2 | 81.82 | 4.01 | 5.23 |
| 3 | 169 | 0 | 62 | 1 | 0 | 5.9 | 5.5 | 154 | 187 | 2 | 79.28 | 2.15 | 7.21 |
| 3 | 170 | 0 | 63 | 1 | 1 | 5 | 5.26 | 142 | 228 | 3 | 81 | 3.92 | 8.79 |
| 3 | 171 | 0 | 71 | 1 | 0 | 5.5 | 5.5 | 154 | 239 | 3 | 83.45 | 9.25 | 19.89 |
| 3 | 172 | 1 | 61 | 1 | 1 | 6.3 | 4.87 | 124 | 267 | 10 | 90.94 | 19.61 | 24.84 |
| 3 | 173 | 0 | 58 | 1 | 1 | 6.3 | 4.56 | 145 | 250 | 2 | 86.09 | 9.23 | 8.3 |
| 3 | 174 | 1 | 69 | 1 | 1 | 6.8 | 5.21 | 133 | 241 | 9 | 91.59 | 6.13 | 15.99 |
| 3 | 175 | 0 | 59 | 1 | 1 | 7.2 | 5.18 | 147 | 267 | 2 | 84.69 | 7.37 | 17.89 |
| 3 | 176 | 0 | 26 | 1 | 1 | 7.5 | 4.9 | 167 | 210 | 3 | 74.07 | 3.15 | 15 |
| 3 | 177 | 0 | 26 | 1 | 1 | 4.6 | 4.6 | 131 | 201 | 3 | 68.25 | 3.14 | 6.56 |
| 3 | 178 | 0 | 27 | 1 | 1 | 4.8 | 4.1 | 120 | 180 | 4 | 88.83 | 3.6 | 9.27 |
| 3 | 179 | 1 | 26 | 1 | 1 | 4.8 | 5.1 | 157 | 289 | 0 | 70.81 | 2.98 | 8.76 |
| 3 | 180 | 1 | 26 | 1 | 1 | 4.8 | 5.2 | 150 | 249 | 4 | 78.51 | 3.42 | 8.07 |
| 3 | 181 | 0 | 25 | 1 | 1 | 5 | 4.6 | 123 | 260 | 3 | 81.9 | 3.34 | 8.63 |
| 3 | 182 | 1 | 25 | 1 | 0 | 5 | 5 | 154 | 145 | 0 | 80.94 | 3.66 | 8.65 |
| 3 | 183 | 1 | 26 | 1 | 0 | 5 | 5.2 | 142 | 173 | 2 | 86.07 | 4.18 | 9.6 |
| 3 | 184 | 1 | 26 | 1 | 0 | 5.5 | 5.4 | 154 | 173 | 4 | 79.99 | 3.85 | 10.88 |
| 3 | 185 | 1 | 28 | 0 | 0 | 6.3 | 5.5 | 153 | 174 | 1 | 71.09 | 3.22 | 9.53 |
| 3 | 186 | 1 | 25 | 1 | 0 | 6.3 | 5.8 | 169 | 220 | 3 | 73.82 | 3.63 | 8.85 |
| 3 | 187 | 1 | 28 | 1 | 0 | 6.8 | 5 | 161 | 175 | 1 | 73.39 | 3.39 | 7.93 |
| 3 | 188 | 1 | 25 | 1 | 0 | 7.2 | 5.1 | 157 | 245 | 0 | 72.26 | 3.1 | 10.69 |
| 3 | 189 | 0 | 26 | 1 | 1 | 7.5 | 4.3 | 138 | 218 | 3 | 65.87 | 3.43 | 12.86 |

Groups: 1 car painting workers, 2 shoes factory workers, 3 the controls.

Gender: 0 female, 1 male. Smoking: 0 smokers, 1 non-smokers. Drinking: 0 alcohol user, 1 non-user.
