# Supplementary material for: The prevalence and persistence of aberrant promoter DNA methylation in benzene-exposed Chinese workers
Source: PLoS One. 2019 Aug 5;14(8):e0220500. doi: 10.1371/journal.pone.0220500 (PMC6681966; doi:10.1371/journal.pone.0220500)
Supplement: S2 Table — (DOCX) [file pone.0220500.s002.docx]

Table S2 The DNA methylation, peripheral blood, and micronuclear frequency of the follow-up painting workers

| Age (year) | work duration  (year) | WBC2013(×10^9^) | RBC2013  (×10^9^) | HB 2013(g/L) | Platelet2013  (×10^9^) | WBC2009  (×10^9^) | HB 2009(g/L) | RBC2009  (×10^9^) | Platelet 2009  (×10^9^) | MN  2009  (‰) | MN  2013  (‰) | LINE1  2013  (%) | MGMT  2013  (%) | MLH1  2013  (%) | LINE1  2009  (%) | MGMT  2009  (%) | MLH1  2009  (%) |
| --- | --- | --- | --- | --- | --- | --- | --- | --- | --- | --- | --- | --- | --- | --- | --- | --- | --- |
| 36 | 10 | 3.3 | 5.14 | 150 | 179 | 2.8 | 124 | 3.76 | 165 | 5 | 5 | 43.61 | 5.74 | 5.46 | 47.61 | 3.41 | 10.64 |
| 31 | 12 | 4.6 | 5.37 | 157 | 163 | 3.1 | 135 | 4.19 | 159 | 2 | 4 | 53.14 | 10.15 | 6.21 | 49.37 | 4.99 | 10.89 |
| 25 | 3 | 3.1 | 5.3 | 152 | 170 | 3.3 | 149 | 4.26 | 162 | 2 | 6 | 44.9 | 5.96 | 6.78 | 41.82 | 3.31 | 7.91 |
| 31 | 10 | 18.1 | 5.53 | 165 | 321 | 3.3 | 124 | 3.31 | 126 | 4 | 6 | 52.76 | 8.57 | 12.56 | 42.39 | 3.71 | 9.3 |
| 31 | 10 | 4.8 | 5.76 | 163 | 125 | 3.4 | 147 | 4.23 | 123 | 5 | 6 | 54.19 | 7.71 | 10.53 | 63.14 | 10.22 | 17.89 |
| 44 | 9.5 | 3.5 | 5.2 | 157 | 198 | 3.5 | 134 | 4.01 | 166 | 8 | 4 | 54.3 | 5.18 | 6.04 | 43.65 | 4.02 | 9.93 |
| 25 | 4 | 3.8 | 5.38 | 150 | 241 | 3.6 | 140 | 4.15 | 223 | 4 | 4 | 51.76 | 6.83 | 10.82 | 42.23 | 6 | 4.92 |
| 29 | 5.5 | 4.3 | 5.33 | 156 | 145 | 3.6 | 147 | 4.76 | 133 | 3 | 2 | 51.44 | 6.05 | 7.08 | 17.31 | 20.28 | 23.96 |
| 34 | 6 | 4.5 | 5.33 | 162 | 218 | 3.6 | 145 | 4.01 | 169 | 2 | 4 | 61.85 | 7.09 | 9.63 | 39.66 | 5.41 | 4.78 |
| 21 | 4 | 5 | 5.68 | 165 | 173 | 3.6 | 133 | 4 | 173 | 2 | 2 | 50.77 | 6.59 | 6.32 | 61 | 10.23 | 5.25 |
| 31 | 7 | 5.1 | 5.35 | 163 | 154 | 3.7 | 128 | 3.76 | 172 | 1 | 1 | 59.33 | 11.86 | 12.87 | 58.29 | 8.7 | 16.14 |
| 37 | 4 | 4 | 4.73 | 131 | 194 | 3.8 | 122 | 3.85 | 163 | 3 | 7 | 53.51 | 5.62 | 9.19 | 44.76 | 6.29 | 5.58 |
| 23 | 4 | 5.3 | 5.02 | 145 | 183 | 3.8 | 129 | 3.84 | 164 | 1 | 1 | 44.75 | 5.69 | 24.99 | 39.66 | 3.29 | 11.48 |
| 28 | 7 | 4 | 4.66 | 134 | 167 | 4 | 130 | 3.72 | 155 | 2 | 5 | 53.71 | 8.99 | 11.29 | 48.46 | 6.66 | 18.76 |
| 26 | 5 | 4.2 | 4.89 | 137 | 191 | 4 | 114 | 3.64 | 178 | 0 | 7 | 34.35 | 7.35 | 5.13 | 34.05 | 2.87 | 5.18 |
| 30 | 10 | 4.8 | 4.91 | 145 | 149 | 4 | 142 | 4.27 | 229 | 1 | 3 | 57.4 | 6.71 | 7.49 | 47.78 | 10.15 | 4.6 |
| 24 | 6 | 3.5 | 4.79 | 135 | 104 | 4.1 | 119 | 3.78 | 218 | 2 | 2 | 43.15 | 6.16 | 7.92 | 37.52 | 3.04 | 12.42 |
| 39 | 6 | 4.2 | 5.54 | 157 | 200 | 4.2 | 141 | 4.31 | 166 | 6 | 3 | 44.59 | 5.92 | 5.64 | 51.84 | 5.69 | 10.81 |
| 26 | 9 | 3.3 | 4.89 | 139 | 123 | 4.3 | 128 | 3.96 | 154 | 1 | 2 | 45.86 | 4.42 | 4.57 | 48.66 | 3.97 | 14.17 |
| 28 | 4 | 4.1 | 5.58 | 170 | 294 | 4.3 | 137 | 3.84 | 239 | 1 | 5 | 45.41 | 4.93 | 7.78 | 44.89 | 4.09 | 4.99 |
| 32 | 10.9 | 4.5 | 5.06 | 151 | 170 | 4.3 | 125 | 3.64 | 163 | 8 | 2 | 55.42 | 8.09 | 11.35 | 53.38 | 3.19 | 13.19 |
| 41 | 7 | 4.8 | 5.57 | 159 | 237 | 4.4 | 139 | 4.69 | 250 | 2 | 2 | 48.85 | 6.23 | 9.16 | 39.53 | 3.03 | 5.95 |
| 27 | 10 | 5 | 5.27 | 157 | 199 | 4.4 | 129 | 3.96 | 128 | 4 | 3 | 64.62 | 6.72 | 13.47 | 51.15 | 6.51 | 12.31 |
| 43 | 4 | 5 | 4.52 | 139 | 80 | 4.4 | 117 | 3.22 | 65 | 8 | 4 | 52.01 | 8.88 | 11.85 | 50.67 | 5.49 | 14.64 |
| 35 | 7 | 5.6 | 5.16 | 147 | 187 | 4.5 | 129 | 3.86 | 160 | 3 | 4 | 43.54 | 4.07 | 4.67 | 47.73 | 3.78 | 14.24 |
| 29 | 7 | 5.1 | 5.19 | 157 | 195 | 4.6 | 143 | 3.98 | 188 | 2 | 1 | 54.59 | 4.77 | 6.36 | 44.19 | 7.6 | 12.64 |
| 22 | 3 | 4.9 | 4.61 | 140 | 191 | 5.6 | 145 | 4.14 | 238 | 3 | 2 | 46.98 | 4.76 | 6.04 | 18.55 | 16.33 | 8.16 |
| 30 | 7 | 8.1 | 6 | 168 | 223 | 5.6 | 165 | 4.77 | 228 | 2 | 6 | 64.15 | 2.92 | 20.19 | 45.12 | 6.6 | 10.22 |
| 22 | 4 | 5.1 | 5.51 | 161 | 151 | 5.9 | 161 | 5.04 | 118 | 2 | 0 | 61.71 | 8.19 | 9.59 | 57.12 | 7.22 | 21.2 |
| 21 | 4 | 5.5 | 5.57 | 166 | 182 | 6.6 | 157 | 4.32 | 192 | 2 | 4 | 60.24 | 8.16 | 8.7 | 46.21 | 6.2 | 10.02 |
| 23 | 3 | 8.1 | 4.79 | 137 | 279 | 6.9 | 164 | 4.51 | 159 | 2 | 3 |  |  |  |  |  |  |
| 43 | 9 | 7.1 | 6 | 122 | 130 | 7.2 | 109 | 4.21 | 230 | 0 | 1 | 48.38 | 5.08 | 5.87 | 43.19 | 6.41 | 9.01 |
| 44 | 10 | 11.7 | 4.97 | 163 | 253 | 7.3 | 142 | 3.77 | 219 | 3 | 6 | 57.86 | 6.96 | 13.06 | 42.99 | 8.71 | 9.83 |
| 32 | 10 | 5.9 | 5.02 | 142 | 292 | 7.4 | 111 | 3.34 | 184 | 2 | 4 | 53.4 | 7.1 | 6.62 | 49.3 | 3.94 | 9.11 |
| 33 | 13 | 4.8 | 5.16 | 158 | 146 | 8.6 | 154 | 4.26 | 222 | 2 | 2 | 32.12 | 6.85 | 10.29 | 39.53 | 3.33 | 24.14 |
