# Supplementary material for: The prevalence and persistence of aberrant promoter DNA methylation in benzene-exposed Chinese workers
Source: PLoS One. 2019 Aug 5;14(8):e0220500. doi: 10.1371/journal.pone.0220500 (PMC6681966; doi:10.1371/journal.pone.0220500)
Supplement: S4 Table — S4a The premotor methylation of MLH1 after the treatment of benzoquinone of AML-5 cells by BSP methods; S4b The premotor methylation of MLH1 after the treatment of hydroquinone of AML-5 cells by BSP methods. (DOCX) [file pone.0220500.s004.docx]

Table S4a The premotor methylation of MLH1 after the treatment of benzoquinone of AML-5 cells by BSP methods

| Number | Mismatch (gap) | Alignment length (% identity) | Methylated CpG (%) | Uncovered (% convered) |
| --- | --- | --- | --- | --- |
| 1 | 0 (0) | 287 (100.0) | 0 (0) | 0/79 (100.0) |
| 2 | 1 (1) | 287 (99.7) | 0 (0) | 2/78 (97.4) |
| 3 | 0 (0) | 287 (100.0) | 0 (0) | 0/79 (100.0) |
| 4 | 2 (0) | 287 (99.3) | 0 (0) | 0/79 (100.0) |
| 5 | 36 (22) | 287 (87.5) | 0 (0) | 1/73 (98.6) |
| 6 | 1 (0) | 287 (99.7) | 0 (0) | 1/79 (98.7) |
| 7 | 0 (0) | 287 (100.0) | 1 (6.2) | 0/79 (100.0) |
| 8 | 1 (1) | 287 (99.7) | 1 (6.2) | 1/78 (98.7) |
| 9 | 7(0) | 287 (97.6) | 1 (6.2) | 0/79 (100.0) |
| 10 | 0 (0) | 287 (100.0) | 2 (12.5) | 1/79 (98.7) |

Bisulfite sequencing PCR (BSP) method was applied to detect premotor methylation of MLH1 of AML-5 cell. 16 CpG inlands were detected. The tenth sequence results were showed in the tables after treatment of benzoquinone.

Table S4b The premotor methylation of MLH1 after the treatment of hydroquinone of AML-5 cells by BSP methods

| Number | Mismatch (gap) | Alignment length (% identity) | Methylated CpG (%) | Uncovered (% convered) |
| --- | --- | --- | --- | --- |
| 1 | 0 (0) | 287 (100.0) | 0 (0) | 0/79 (100.0) |
| 2 | 2 (1) | 287 (99.3) | 0 (0) | 0/78 (100.0) |
| 3 | 2 (0) | 287 (99.3) | 0 (0) | 1/79 (98.7) |
| 4 | 2 (0) | 287 (100.0) | 0 (0) | 1/79 (98.7) |
| 5 | 0(0) | 287 (100.0) | 0 (0) | 1/79 (98.7) |
| 6 | 1 (0) | 287 (99.7) | 0 (0) | 1/79 (98.7) |
| 7 | 0 (0) | 287 (100.0) | 0 (0) | 5/79 (93.7) |
| 8 | 0 (0) | 287 (100.0) | 0 (0) | 2/79 (97.5) |
| 9 | 0(0) | 287 (100.0) | 0 (0) | 0/79 (100.0) |
| 10 | 0 (0) | 287 (100.0) | 0 (0) | 1/79 (98.7) |

Bisulfite sequencing PCR (BSP) method was applied to detect premotor methylation of MLH1 of AML-5 cell. 16 CpG inlands were detected. The tenth sequence results were showed in the tables after treatment of hydroquinone.
